# Charting the Path: Psychological Factors and Diet Adherence in Adolescents With Celiac Disease

**DOI:** 10.7759/cureus.74103

**Published:** 2024-11-20

**Authors:** Antonios Bozas, Georgios Karakatsoulis, Efharis Panagopoulou, Ioannis Xinias, Maria Fotoulaki

**Affiliations:** 1 4th Department of Pediatrics, Aristotle University of Thessaloniki, Thessaloniki, GRC; 2 Institute of Applied Biosciences, Center for Research and Technology Hellas (CERTH), Thessaloniki, GRC; 3 Laboratory of Primary Health Care, Aristotle University of Thessaloniki, Thessaloniki, GRC; 4 3rd Department of Pediatrics, Aristotle University of Thessaloniki, Thessaloniki, GRC

**Keywords:** celiac disease, developmental transition, dietary compliance, gluten adherence, psychological elements

## Abstract

Introduction

Adolescence is a pivotal time for individuals with celiac disease (CD), presenting a host of psychosocial challenges. Managing a strict gluten-free diet (GFD) while forming self-identity, striving for autonomy, and navigating social relationships significantly impacts adolescents with CD. The present pilot study investigates the impact of psychological factors on behavioral and dietary responses in adolescents with CD, utilizing repeated measures over time.

Methods

Thirty-one adolescents aged 11-18 from a pediatric outpatient CD department were recruited. Participants completed the Resilience Youth Development Module (RYDM), the Child and Adolescent Mindfulness Measure (CAMM), the Strengths and Difficulties Questionnaire (SDQ), and Celiac Dietary Adherence Test (CDAT) over four phases spanning four months. Twenty-seven parents also agreed to participate in completing the SDQ parents’ version.

Results

The results revealed moderate levels of mindfulness and resilience, accompanied by inadequate adherence to a GFD. Baseline assessments revealed difficulties in peer relationships and psychosocial functioning, which parents corroborated. Over time, reductions in mindfulness and resilience were observed, along with modest improvements in dietary adherence and decreases in psychosocial difficulties.

Discussion

This study underscores the importance of psychological traits, suggesting that enhancing mindfulness and resilience may improve both dietary adherence and overall well-being. However, given the dynamic nature of adolescents’ coping strategies through developmental changes and social environments, tailored interventions are essential for effective disease management and social integration.

## Introduction

Celiac disease (CD) is identified as an autoimmune disorder that is triggered by the ingestion of gluten, causing damage to the small intestine and impairing nutrient absorption. Approximately 1% of the general population presents celiac manifestation with higher rates, mainly in Western Countries (Europe and North America) [1]. Compared to adults, children and adolescents are at twice the risk of being diagnosed, while this is mainly connected with developmental deficits, which can be more easily observed in childhood. Moreover, first-degree relatives of individuals with CD have a 10-15% likelihood of developing symptomatology [1,2].

Adolescents with CD face the complex challenge of balancing their developmental need for autonomy and identity formation with strict gluten avoidance. Dietary restrictions can influence their behavior, emotions, and well-being [1]. The transitional period to adulthood, characterized by the need for autonomy and acceptance from others, goes through strict vigilance to a gluten-free diet (GFD). As a result, adolescents tend to develop emotional difficulties and social restrictions, somehow leading to maladaptive eating behaviors [1,2]. Not only are they vulnerable to food-related anxiety, but they are also at risk of eating-related issues and compulsiveness linked with food consumption. Behavioral responses such as avoidance, control, and rigidity are observed and thought to occur because of adhering to a GFD [1,2].

These dietary restrictions often lead adolescents to feel different from others or isolate themselves, affecting their self-esteem and social participation. Peer pressure can exacerbate these issues, in contexts like school or teen parties centered around food. As a result, they might face stigmatization, leading to either withdrawal or hiding their condition, both affecting the non-adherence to the GFD [3]. So their ability to integrate their condition into their daily life and how they identify themselves became even more complicated [4].

Moving to adulthood gradually shifts the responsibility of managing the condition from parents to adolescents. This transition can profoundly impact adherence to a GFD, including challenges that can influence compliance. Moreover, it can affect well-being, quality of life (QoL), and maintenance of healthy eating habits [2,5]. This is particularly important as adolescents transition into adulthood, where independence and social integration become increasingly vital [3,6].

On that note, there is a range of strategies, from adaptive to maladaptive, that directly affect the response to disease management and self-regulation [7,8]. Characteristics like flexibility, self-trust, mindful attention to food consumption, and resilience are linked to adaptive strategies adopted by adolescents. On the contrary, dietary rigidity, compulsive response to food and strict constraints, and reduced social interaction are some of the maladaptive strategies that have been observed in this population [7,8].

Resilience and mindfulness can play a critical role in how adolescents cope with the challenges posed by CD. The ability to adapt to the dietary restrictions and social implications of CD can mitigate some of the psychological burdens associated with the condition. Research indicates that adolescents who develop strong coping strategies and resilience are better equipped to manage their health condition and maintain adherence [9,10]. Adolescents with higher resilience levels seem to be able to manage their health and prevent complications. Furthermore, mindfulness practices can enhance resilience by promoting emotional regulation and reducing anxiety, allowing adolescents to navigate their social environments more effectively [11,12]. Mindfulness practices encourage present-moment awareness and acceptance, alleviating anxiety related to food choices and social situations.

This pilot study tries to approach how psychological factors, specifically resilience and mindfulness, can influence dietary adherence among adolescents with CD. This primary investigation is expected to identify how better levels of resilience and mindfulness can contribute to better GFD adherence, while better psychosocial outcomes will also support this. These psychological factors might also fluctuate during adolescence when social pressure and emotional challenges are increasing. However, adolescents presenting better psychosocial functioning are expected to be more consistent with dietary requirements. Furthermore, parents’ perception of their children’s psychosocial functioning will correlate with their adherence to GFD, indicating that insights coming from them may serve as a valuable predictor of dietary compliance. The idea of conducting assessments over time was made to capture the dynamic nature of resilience and mindfulness and how these evolve dietary adherence. This short-term longitudinal approach allows us to observe changes in coping mechanisms and dietary patterns in response to the developmental period. Primary findings can offer an understanding of how relevant factors can influence long-term treatment, while they will contribute and provide guidance to future, larger-scale research on the psychological dynamics affecting CD management in adolescents.

## Materials and methods

Participants and criteria

The study design and protocol were ethically approved by the Bioethics Committee of the Aristotle University of Thessaloniki, Thessaloniki, Greece. Participants aged 11-18 with a confirmed biopsy diagnosis of CD and the European Society for Pediatric Gastroenterology Hepatology and Nutrition (ESPGHAN) criteria were included [13]. Individuals with the following criteria were excluded from the study: (i) experience of acute disease, (ii) co-existence of another GI, (iii) neurodevelopmental disability or chromosomal abnormality, (iv) psychiatric comorbidity, and (v) age less than 11 or young adults (18<). The total number of participants who agreed and completed all phases of the study was 31. Participants were adolescents, all coming from the Northern Greece region and followed the official Greek educational system. The sample reflects all different social classes as the unit is a public health university unit covering all beneficiaries who were referred. The total number of parents who also participated in the final phase of the study was 27 (one common parent for two adolescents and three who refused to complete the psychometric assessment). Additional demographic information was collected during the recruitment phase. Participants were assessed on a set of psychological elements, including psychosocial functioning and compliance with gluten consumption. Parents of patients were also asked to report the adolescents’ psychosocial functioning questionnaire (parents’ version).

Data collection

Study data was collected between December 2022 and December 2023. Informative letters were shared, and participants entered the study only after the written informed consent had been received. The data collection process took place in the following three phases: during the first phase (recruitment), a baseline assessment, including disease-related medical history, demographics, and levels of resilience (Resilience Youth Development Module [RYDM]), took place. The second phase included a follow-up study through a structured diary. Participants were asked to complete that diary for four months (once per month). This follow-up assessment, four repeated measures, included self-reported measures that evaluated the following: (i) dietary adherence (Celiac Adherence Scale), (ii) psychosocial functioning (Strengths and Difficulties Questionnaire [SDQ]), and (iii) attention to the present moment (Child and Adolescent Mindfulness Measure [CAMM]). In the third and final phase, after the completion of the repeated measures, individuals were asked to provide a follow-up assessment of their resilience. Parents of participants also completed the SDQ parents’ version.

Psychometric measurements

Resilience Youth Development Module (RYDM)

The RYDM is a self-report measure that aims to evaluate protective resilience factors. The Greek version assesses internal and external factors of resilience (self-trust/self-knowledge, goals and ambitions, adult support outside the family, peer support, school support, and home-family support) [14,15].

Strength and Difficulties Questionnaire (SDQ)

SDQ-child version: SDQ is a self-report measure that provides a behavioral screening regarding the psychosocial functioning of the target population. There are five subscales (emotional symptoms, conduct problems, hyperactivity/inattention, peer relationship problems, and prosocial behavior) and a total score (total difficulty). SDQ is widely used in different settings and with satisfactory psychometric properties [16].

SDQ-parent version: The SDQ-parent version is the second of three versions (child-teacher-parent) and consists of the same subscales. It can assess behavioral aspects from both perspectives, thus increasing the reliability of the existence of reported difficulties [16,17].

Celiac Dietary Adherence Test (CDAT)

CDAT is a seven-item questionnaire designed to measure the adherence of celiac patients. The questionnaire is primarily designed for adults but can also be used for adolescents. It uses a Likert scale from 0 to 5 and points scores from 7 to 35. Scores less than 13 indicate good adherence to the GFD, and scores above 17 indicate poor adherence [18].

CAMM

CAMM is a 10-item self-reported measure that assesses awareness of the present moment and non-judgmental attitudes. It is structured in a one-dimensional factor (5-point Likert scale). Higher scores are up to 40. Lower scores indicate higher tendencies to a mindful position toward daily life [19,20].

The Greek versions of the questionnaires used in this study, including RYDM, SDQ, CDAT, and CAMM, are provided in the Appendix.

Statistical analysis

In descriptive statistics, frequencies and relative frequencies were used for the categorical variables, whereas mean and standard deviation were used for the numeric. For inferential statistics, the Pearson correlation coefficient was used to examine the correlations between the scales, while the behavior of scale scores over time was investigated through paired t-test (if only measured twice) and repeated measures ANOVA (if measured more than twice). The significance level was set at 5%, and all the comparisons were two-tailed. All statistical analyses were conducted in R (version 4.3.2).

## Results

Demographics and clinical information

The sample included 31 pediatric patients, with 19 (61.3%) females. The mean age was 13.6 ± 2.06 years, with 17 (54.8%) being symptomatic. Six (19.4%) reported a psychosocial history and no diagnosis (anxiety-related issues, ADHD, and emotional disorders). Furthermore, 12 (38.7%) reported relevant family history, and five (16.1%) presented comorbidities (Hashimoto thyroiditis and food allergies), whereas six (19 %) were under treatment (mainly for allergies or for ferritin and vitamin D deficits). The total number of parents who participated was 27.

Baseline values and follow-up measurements

In this section, baseline values for the subscales of each instrument are described. For simplicity, the mean and standard deviation are only being reported (see Table 1). The assessment revealed moderate levels of mindfulness and resilience, paired with suboptimal adherence to a GFD. With regards to mindfulness (CAMM), the sample’s average score (M = 17.52, SD = 7.05) suggested a moderate capacity to remain mindful. This could reflect an awareness of dietary challenges but highlights a potential gap in emotional regulation strategies. CDAT scores (M = 18.06, SD = 3.92) indicate an inadequate GFD adherence in the current sample that aligns with the literature, suggesting that adolescents with CD face significant barriers to strict dietary compliance due to developmental transitions and/or social factors. Participants also, as mentioned above, displayed moderate levels of resilience (RYDM; M = 64.48, SD = 15.62), with variability across subscales like peer support, school support, and self-trust. It could be indicated that while protective factors like peer and family support can be in place, they are still facing notable resilience gaps, potentially affecting their capacity and copying to CD-related stressors. When it comes to psychosocial challenges, some are highlighted with particular interest in peer relationships (SDQ; M = 5.39, SD = 2.03). The additional parent-reported scores mirrored relevant findings, suggesting a consensus between parents and adolescents on the social struggles associated with CD, such as feeling isolated or different in food-centered social activities (see Table 1).

**Table 1:** Means and standard deviations of scales CAMM, Child and Adolescent Mindfulness Measure; CDAT, Celiac Dietary Adherence Test; SDQ, Strengths and Difficulties Questionnaire; SDQ subscales (both versions) ADHD, hyperactivity/inattention; CD, conduct problems; PR, peer relationship problems; EB, emotional problems; PSB, prosocial behavior; RYDM, Resilience Youth Development Module; RYDM subscales, self-trust, goals, empathy, adult support, peer support, school support, and home family

Variables	M (SD)
CAMM	17.52 (7.05)
CDAT	18.06 (3.92)
SDQ children (total)	15.23 (4.96)
ADHD	4.81 (1.3)
BD	3.32 (1.92)
CD	3.58 (2.06)
PR	3.52 (1.81)
PSB	5.39 (2.03)
RYDM (total)	64.48 (15.62)
Self-trust	10.45 (2.63)
Goals	8.45 (2.5)
Empathy	5.55 (1.88)
Adult support	11.39 (4.01)
Peer support	9.58 (3.01)
School support	9.16 (3.13)
Home family	9.9 (2.7)
SDQ parents	14.04 (4.31)
ADHD	4.04 (1.45)
CD	2.67 (2)
EB	3.44 (1.85)
PRB	3.89 (1.55)
PS	3.67 (1.07)

Behavior of scale scores over time

The behavior of the CAMM, CDAT, SDQ (see Table 2), and RYDM (see Table 3) questionnaires are examined over time. Across the four assessments, key shifts in mindfulness, dietary adherence, and psychosocial functioning emerged, suggesting coping strategies evolved over time. A notable reduction in mindfulness scores was observed over time (β = −0.91, 95% CI [−1.48, −0.35], p = 0.002). This might indicate a diminishing ability to remain self-aware, potentially due to the increasing emotional demands of managing CD. Reduced mindfulness might reflect adolescents’ shifting focus from internal emotional regulation to external compliance with dietary restrictions. Dietary adherence scores were found to have improved modestly over time (β = −0.51, 95% CI [−0.9, −0.11], p = 0.014), presenting gradual enhancement in dietary compliance. The more experience adolescents gain through their effort to manage their diet and the social boundaries they face, the more they accept the dietary limitations and the fact that this will be a lifelong condition. However, the improvement remains modest, suggesting ongoing adherence challenges. With regards to psychosocial functioning, SDQ scores presented a decline (β = −0.93, 95% CI [−1.32, −0.55], p < 0.001), particularly in subdomains related to hyperactivity and behavioral difficulties. A decrease in ADHD-related behaviors (β = −0.45, p < 0.001) and general conduct issues (β = −0.31, p = 0.003) suggests an improvement in behavioral self-regulation over time. Reductions in peer problems, however, were less pronounced, suggesting that social integration remains a critical area (see Table 2). Resilience (RYDM) was measured twice during the study period, with a modest but notable reduction in scores from the initial to the second assessment point (Cohen’s d = −0.438, p = 0.025). Specific subscales demonstrated significant declines, suggesting that adolescents perceived less personal and social support over time. More specifically, goals present a significant decline (Cohen’s d = −0.736, p < 0.001), which might link to less drive or ability to set personal objectives. Home family support scores are also decreased, implying a reduced perception of family support as adolescents navigated dietary and social challenges associated with CD. Empathy and peer support present modest reductions, indicating minor declines in social connectedness and emotional responsiveness (see Table 3).

**Table 2:** CAMM, CDAT, and SDQ child (sub)scales over time CAMM, Child and Adolescent Mindfulness Measure; CDAT, Celiac Dietary Adherence Test; SDQ, Strengths and Difficulties Questionnaire; PR, peer relationship problems; PSB, prosocial behavior

Variable	1	2	3	4	β (95% CI)	p-value
CAMM	17.52 (7.05)	16.61 (5.23)	15.65 (4.99)	15.16 (5.15)	-0.91 (-1.48, -0.35)	0.002
CDAT	18.06 (3.92)	16.84 (2.99)	16.42 (2.55)	16.61 (3.09)	-0.51 (-0.9, -0.11)	0.014
SDQ	15.23 (4.96)	14.26 (3.25)	13.19 (2.68)	12.68 (3.15)	-0.93 (-1.32, -0.55)	<0.001
ADHD	4.81 (1.3)	3.52 (1.46)	3.68 (1.17)	3.23 (1.41)	-0.45 (-0.64, -0.26)	<0.001
BD	3.32 (1.92)	3.77 (1.75)	2.97 (1.25)	2.77 (1.38)	-0.31 (-0.51, -0.12)	0.003
CS	3.58 (2.06)	3.32 (1.38)	2.97 (1.25)	3.26 (1.24)	-0.13 (-0.34, 0.08)	0.237
PR	3.52 (1.81)	3.65 (1.28)	3.58 (1.09)	3.42 (1.2)	-0.04 (-0.24, 0.17)	0.734
PSB	5.39 (2.03)	3.84 (2)	3.87 (1.52)	4.19 (1.76)	-0.33 (-0.58, -0.08)	0.011

**Table 3:** RYDM scores first and second assessments RYDM, Resilience Youth Development Module; subscales of RYDM, self-trust, goals, empathy, adult support, peer support, school support, and home family

Variable	1	2	Cohen’s d	p-value
RYDM	64.48 (15.62)	56.23 (12.36)	-0.438	0.012
Self-trust	10.45 (2.63)	9.74 (2.86)	-0.16	0.267
Goals	8.45 (2.5)	6.48 (1.77)	-0.736	<0.001
Empathy	5.55 (1.88)	4.71 (1.19)	-0.373	0.036
Adult support	11.39 (4.01)	10.58 (2.51)	-0.092	0.288
Peer support	9.58 (3.01)	8.26 (1.9)	-0.379	0.029
School support	9.16 (3.13)	8.45 (2.59)	-0.17	0.282
Home family	9.9 (2.7)	8 (2.13)	-0.615	<0.001

Correlations between the scales

The correlation analysis highlighted further interdependencies between traits and dietary adherence, with particular findings presented below (Table 4).

**Table 4:** Correlations between scales Correlations marked with * indicate significance at p < 0.05; correlations marked with ** indicate significance at p < 0.01. CAMM, Child and Adolescent Mindfulness Measure; CDAT, Celiac Dietary Adherence Test; RYDM, Resilience Youth Development Module; SDQ, Strengths and Difficulties Questionnaire

Variable	CAMM	CDAT	RYDM	SDQ
CAMM	1			
CDAT	0.64**	1		
RYDM	0.28	0.47**	1	
SDQ	0.7**	0.58**	0.36*	1

Mindfulness and Dietary Adherence

CAMM scores correlated positively with CDAT (r = 0.64, p < 0.01), suggesting that higher mindfulness is associated with better adherence to GFD. The idea that mindful awareness can enhance dietary regulation is supported by the possibility of reinforcing attentiveness to food choices and reducing impulsive deviations from GFD.

Mindfulness and Psychosocial Functioning

CAMM also positively correlated with SDQ scores (r = 0.7, p < 0.01), indicating that greater mindfulness is linked to fewer reported psychosocial difficulties. VC indeed, mindful engagement can mitigate social and emotional challenges, helping adolescents navigate interpersonal stresses related to their condition.

Resilience and Adherence

Correlations between RYDM and CDAT are found to be moderate (r = 0.47, p < 0.01), suggesting that resilience also plays a role in dietary compliance. Interestingly, resilience also showed a small but notable correlation with SDQ scores (r = 0.36, p < 0.05), implying that resilient adolescents may face fewer behavioral challenges in adapting to their condition.

## Discussion

The present pilot study aims to explore the interrelationships and the temporary dynamics among psychological elements, behavioral responses, and dietary adherence in an adolescent sample population targeting to contribute to the current literature on the connection between dietary adherence and well-being.

Interesting insights reveal that the present sample is characterized by moderate levels of mindfulness and resilience (baseline analysis) and insufficient adherence to GFD. Furthermore, there are slightly raised tendencies to difficulties with peer problems, which are reported as a challenge, as confirmed by parents’ reports as well. Resilience levels were found to be moderate, while reported scores suggest adolescents’ reduced capability to be mindfully engaged in their food behavior and less likely to be aware of the possibility of consuming gluten. Findings further support the role of both psychological elements as predictors for health and well-being [21,22]. Insufficient adherence to GFD as a health outcome is directly affected by the resilience capacity and the ability to stay present. Both respond to disease-related stressors and mediate the way stress is expressed. Moreover, these are consistent with the literature on chronic condition management, where acceptance of the experience and capacity to overcome daily challenges promotes better adherence and provides better outcomes. In contrast, the absence of this capacity works in reverse [21-23].

Psychosocial reported outcomes are also in line with a wide range of studies suggesting that adolescents with CD are at risk of manifesting emotional difficulties. At the same time, social interaction remains the main challenge [2]. Peer relationships and social interaction remain a challenge for adolescents with CD as it separates them from their social groups during a period where the sense of belonging is crucial [24,25]. Good peer relationships play a crucial role during this transitional period, affecting the coping strategies adolescents adopt to overcome a burden. Parents’ reports also reveal relevant worries about their children’s social difficulties. Parents express worries about managing food restrictions, coping with raised financial burdens, and their need to cover the actual social burden their children are facing [26-28].

Regarding mindfulness and resilience, several literature findings suggest that both factors seem to affect well-being and general health through their mediation role in adopting relevant copying strategies, while they can either enhance or weaken an individual’s capacity to be able to bounce back and, despite the challenges be able to achieve better health outcomes [11,12]. Staying away from gluten, especially when it comes to adolescents, is a challenge to follow and be aware of (mindful) but also to develop strategies to continue to exist in a context where although gluten exists, he/she still adopts a healthy to him/herself response (resilience). As a result, also observed in the sample, the achievement or not in avoiding gluten consumption shapes the emotional and behavioral responses relatively, so either psychosocial functioning presents growth or reduction [12,29,30]. These behavioral repertoires include nuances of psychological elements. Based on the current sample, while levels of mindfulness and resilience are reported to be lower, SDQ and CDAT scores are higher. That means better-reported adherence to celiac dietary and less expressed psychosocial difficulties over time.

Although improved adherence leads to better health outcomes, psychological factors like mindfulness and resilience can be declined. This “paradox” can be explained by taking into consideration the emotional and cognitive burden of keeping a strict diet. They can shift their copying strategies to focus on controlling the dietary intake at the expense of broader emotional regulation and stress management [3,31]. A decline in the ability to remain present and engaged thoughtfully could reflect their focus on rigid compliance with dietary rules rather than a balanced, mindful approach to living with CD and affect their resilience. On that note, dietary compliance can alleviate symptoms, but it does not necessarily address the distress and burden that many adolescents experience with their condition [2].

Their essential need is to establish autonomy and competence. This ongoing process is dynamic, so designed scales capturing the moment might not be able to highlight those nuances of the process [31]. Adolescents moving gradually to early adulthood are passing through several developmental changes by developing skills and mechanisms to manage daily challenges. Coping strategies are a constantly growing process, affected by new interests, social competency, or new priorities. Thus, several advances might need more time to be reflected in mindfulness states [31].

On the other hand, resilience scores reflect this multi-facility construct, which encompasses interpersonal resources and individual characteristics that affect copying. Building resilience comes as a response to challenges that gradually change during adolescence [32]. What is considered a resilient response at a specific time point does not surely respond to a newly emerged stressor. And this is also connected with related developmental advances. In the current sample, reduced resources, such as personal goals, family relationships, and empathy, affect the general resilience levels [32]. However, chronic conditions somehow direct the responses gradually into a more adaptive way. That might explain the fact that although the general resilience could be slightly reduced, adolescents still avoid gluten [30].

Lastly, personal characteristics and identity elements could influence individual copying. Early attitudes or personality characteristics can direct a response. In a health condition, copying is a complex process influenced by several factors [3,4]. This ongoing process meets the identity shaping so easily that it can affect behavioral patterns adopted at that point and in early adulthood as well. Bringing the focus on identifying the different early influences in health behavioral responses could also provide interesting information in understanding the structure of illness-related identity. And that could also lead to individualized and professional interventions in supporting adolescents [3,4].

Limitations and future implications

While this pilot study provides valuable insights into the relationship between psychological traits and diet adherence in adolescents with CD, several limitations should be noted. The small sample size and short follow-up period limit the generalizability of the findings to broader populations. Additionally, the reliance on self-reported measures introduces the possibility of response bias, particularly in assessments of dietary adherence and psychosocial functioning. Thus, extended research will help clarify whether these changes persist or evolve as adolescents mature. Additionally, investigating individual differences in coping strategies and social support systems could inform personalized interventions that better address the emotional and social challenges of chronic disease management.

Future studies, based on the current and relevant pilots, with larger, more diverse samples and longer follow-up periods, are important to validate these findings and better understand the long-term effects of resilience and mindfulness on disease management in adolescents with CD. Future research should also focus on testing mindfulness-based interventions and resilience training specifically designed for adolescents, aiming to improve both psychological well-being and adherence to a GFD. Including family-based approaches could further strengthen these interventions, given the role of parental support in disease management. Overall, this study sets the stage for more comprehensive efforts to enhance both the psychological and practical aspects of chronic illness care in adolescents.

## Conclusions

This pilot study tries to provide insight into the complex interactions between psychological traits, such as resilience and mindfulness, and chronic disease management in adolescents with CD. By examining their influence on GFD, this study further underscores the critical role of emotional and social functioning in managing CD during adolescence. The findings suggest that while adolescents may show improved dietary adherence, reductions in mindfulness and resilience indicate ongoing psychological challenges. This dynamic interplay highlights the need for more holistic disease management strategies that also focus on adherence and support psychological well-being.

The longitudinal approach allows us to observe how, during this developmental period, health-related behaviors are being affected by different factors. To better understand the impact of psychosocial factors on dietary adherence, it was necessary to use validated tools to enhance the findings’ reliability. Taking into clinical consideration these real-world outcomes, it emphasizes the importance of tailored interventions that foster resilience and mindfulness, helping adolescents to navigate the emotional and social complexities of living with CD. Thus, this study contributes to understanding how psychological traits can shape chronic disease management, emphasizing the need for adherence and well-being.

## Appendices

**Table 5:** Strength and Difficulties Questionnaire (SDQ-parent version) The SDQ-parent version is the second of three versions (child-teacher-parent) and consists of the same subscales. It can assess behavioral aspects from both perspectives, thus increasing the reliability of the existence of reported difficulties [16,17]. The questionnaire is under a Creative Commons License available at sdqinfo.org

	Not True	Somewhat True	Certainly True
Considerate of other people's feelings			
Restless, overactive, and cannot stay still for long			
Often complains of headaches and stomach-aches or sickness			
Shares readily with other children (treats, toys, pencils, etc.)			
Often has temper tantrums or hot tempers			
Rather solitary and tends to play alone			
Generally obedient and usually does what adults request			
Many worries and often seem worried			
Helpful if someone is hurt, upset, or feeling ill			
Constantly fidgeting or squirming			
Has at least one good friend			
Often fights with other children or bullies them			
Often unhappy, down-hearted, or tearful			
Generally liked by other children			
Easily distracted and concentration wanders			
Nervous or clingy in new situations and easily loses confidence			
Kind to younger children			
Often lies or cheats			
Picked on or bullied by other children			
Often volunteers to help others (parents, teachers, and other children)			
Thinks things out before acting			
Steals from home, school, or elsewhere			
Gets on better with adults than with other children			
Many fears and easily scared			
Sees tasks through to the end and has good attention span			

**Table 6:** Celiac Dietary Adherence Test (CDAT) CDAT is a 7-item questionnaire designed to measure the adherence of celiac patients [18]. Permission for the Greek version was obtained from the publisher for the reproduction of this questionnaire. GFD, gluten-free diet

Question	Score				
	1	2	3	4	5
Have you been bothered by low energy levels during the past 4 weeks?	None of the time	A little of the time	Some of the time	Most of the time	All of the time
Have you been bothered by headaches during the past 4 weeks?	None of the time	A little of the time	Some of the time	Most of the time	All of the time
I am able to follow a GFD when dining outside my home	Strongly agree	Somewhat agree	Neither agree nor disagree	Somewhat disagree	Strongly disagree
Before I do something, I carefully consider the consequences	Strongly agree	Somewhat agree	Neither agree nor disagree	Somewhat disagree	Strongly disagree
I do not consider myself a failure	Strongly agree	Somewhat agree	Neither agree nor disagree	Somewhat disagree	Strongly disagree
How important to your health are accidental gluten exposures?	Very important	Somewhat important	Neutral/unsure	A little important	Not at all important
Over the past 4 weeks, how many times have you eaten foods containing gluten on purpose?	0 (never)	1-2	3-5	6-10	>10

**Table 7:** Resilience Youth Development Module (RYDM) The RYDM is a self-report measure that aims to evaluate protective resilience factors [14,15]. Permission for the Greek version was obtained from the publisher for the reproduction of this questionnaire.

Resilience Youth Development Module
Directions: How true do you feel that these statements are about you personally?
(1 = not at all true, 2 = a little true, 3 = pretty much true, 4 = very much true)
I can work with someone who has different opinions than mine.
I can work out my problems.
I can do most things if I try.
There are many things that I do well.
I feel bad when someone gets their feelings hurt.
I try to understand what other people go through.
I try to understand how other people feel and think.
There is a purpose to my life.
I understand my moods and feelings.
I understand why I do what I do.
At my school, there is a teacher or some other adult...
(1 = not at all true, 2 = a little true, 3 = pretty much true, 4 = very much true)
Who really cares about me.
Who tells me when I do a good job.
Who notices when I’m not there.
Who always wants me to do my best.
Who listens to me when I have something to say.
Who believes that I will be a success.
I have a friend about my age who truly cares about me.
I have a friend about my age who talks to me about my problems.
I have a friend about my age who helps me when I'm going through tough times.
My friends try to do the right thing.
My friends do well in school.
At home, at least one of my parents or another adult (who lives with us) expects me to follow the rules.
At home, at least one of my parents or another adult (who lives with us) is interested in my school studies.
At home, at least one of my parents or another adult (who lives with us) believes that I will do well and that I will succeed.
At home, at least one of my parents or another adult (who lives with us) always wants me to do my best.
At home, at least one of my parents or another adult (who lives with us) listens when I have something to say.
Τέλος φόρμας

**Table 8:** Child and Adolescent Mindfulness Measure (CAMM) CAMM is a 10-item self-reported measure that assesses awareness of the present moment and non-judgmental attitudes [19,20]. Permission for the Greek version was obtained from the publisher for the reproduction of this questionnaire.

	Never true	Rarely true	Sometimes true	Often true	Always true
I get upset with myself for having feelings that don’t make sense.	0	1	2	3	4
At school, I walk from class to class without noticing what I’m doing.	0	1	2	3	4
I keep myself busy so I don’t notice my thoughts or feelings.	0	1	2	3	4
I tell myself that I shouldn’t feel the way I’m feeling.	0	1	2	3	4
I push away thoughts that I don’t like.	0	1	2	3	4
It’s hard for me to pay attention to only one thing at a time.	0	1	2	3	4
I get upset with myself for having certain thoughts	0	1	2	3	4
I think about things that have happened in the past instead of thinking about things that are happening right now.	0	1	2	3	4
I think that some of my feelings are bad and that I shouldn’t have them	0	1	2	3	4
I stop myself from having feelings that I don’t like.	0	1	2	3	4

**Table 9:** Strength and Difficulties Questionnaire (SDQ-single version) SDQ is a self-report measure that provides a behavioral screening regarding the psychosocial functioning of the target population. There are five subscales and a total score [16]. The questionnaire is under a Creative Commons License available at sdqinfo.org

	Not true	Somewhat true	Certainly true
I am restless; I cannot stay still for long			
I get a lot of headaches and stomach-aches or sickness			
I usually share with others (food, games, pens, etc.)			
I get very angry and often lose my temper			
I am usually on my own; I generally play alone or keep to myself			
I usually do as I am told			
I worry a lot			
I am helpful if someone is hurt, upset, or feeling ill			
I am constantly fidgeting or squirming			
I have one good friend or more			
I fight a lot; I can make other people do what I want			
I am often unhappy, down-hearted, or tearful			
Other people my age generally like me			
I am easily distracted; I find it difficult to concentrate			
I am nervous in new situations; I easily lose confidence			
I am kind to younger children			
I am often accused of lying or cheating			
Other children or young people pick on me or bully me			
I often volunteer to help others (parents, teachers, and children)			
I think before I do things			
I take things that are not mine from home, school, or elsewhere			
I get on better with adults than with people my own age			
I have many fears; I am easily scared			
I finish the work I'm doing; my attention is good			
